# Case Report: Semantic Variant of Primary Progressive Aphasia Associated With Anti-Glial Fibrillary Acid Protein Autoantibodies

**DOI:** 10.3389/fimmu.2021.760021

**Published:** 2022-01-03

**Authors:** Niels Hansen, Winfried Stöcker, Jens Wiltfang, Claudia Bartels, Kristin Rentzsch, Caroline Bouter

**Affiliations:** ^1^ Department of Psychiatry and Psychotherapy, University Medical Center Goettingen, Goettingen, Germany; ^2^ Euroimmun Reference Laboratory, Luebeck, Germany; ^3^ German Center for Neurodegenerative Diseases (DZNE), Goettingen, Germany; ^4^ Neurosciences and Signaling Group, Institute of Biomedicine (iBiMED), Department of Medical Sciences, University of Aveiro, Aveiro, Portugal; ^5^ Department of Nuclear Medicine, University Medical Center Göttingen, Goettingen, Germany

**Keywords:** frontotemporal lobar degeneration (FTLD), semantic variant of primary progressive aphasia (svPPA), autoimmunity, anti-GFAP antibody, immunotherapy

## Abstract

**Background:**

Frontotemporal lobar degeneration is a heterogeneous disorder entailing a semantic variant of primary progressive aphasia (svPPA). A subtype of frontotemporal dementia associated with glutamate receptor subunit 3 (GluA3) antibody of the α-amino-3-hydroxy-5-methyl-4-isoxazolepropionic acid receptor (AMPAR) was recently identified. Here, we describe the novelty of a svPPA associated with anti-glial fibrillary acid protein (GFAP) antibodies.

**Methods:**

To diagnose this 68-year-old woman we conducted a clinical examination, neuropsychological testing, CSF analysis, MRI and 18F-fluorodeoxyglucose (18F-FDG) Positron Emission Tomography (PET)/computed tomography (CT) imaging.

**Results:**

The clinical phenotype corresponds to a svPPA based on impaired confrontation naming and single-word comprehension. In addition, we observed spared speech production, impaired object knowledge, and surface dyslexia - further supporting the diagnosis of svPPA. Additional characteristic imaging features such as anterior temporal hypometabolism in 18F-FDG PET/CT confirmed patient’s svPPA diagnosis. CSF analysis revealed signs of axonal degeneration, as both tau and phosphorylated tau proteins exceeded normal levels. Her serum showed anti-GFAP autoantibodies.

**Conclusion:**

We diagnosed a svPPA in this patient and report an association between serum anti-GFAP antibodies and svPPA never reported in the literature so far, thereby expanding the clinical spectrum of svPPA and anti-GFAP-antibody related disease. Further research is needed to elucidate the underlying immunopathology of this disease entity to ultimately improve treatment.

## Introduction

Frontotemporal lobar degeneration is a debilitating neurodegenerative disease of heterogeneous etiology. An autoimmune origin was recently postulated in a subgroup of frontotemporal lobar degeneration, most commonly in frontotemporal dementia’s behavioral variant with antibodies directed against the glutamate receptor subunit 3 (GluA3) of the α-amino-3-hydroxy-5-methyl-4-isoxazolepropionic acid receptor (AMPAR) (1, 2). Reports suggest that GFAP autoantibodies are detected in association with dementia, like senile dementia caused by Alzheimer´s and vascular dementia (3). Another study (4) depicted *via* immunoassay that patients with Alzheimer´s disease have higher levels of anti-GFAP autoantibodies. However, no GFAP autoantibodies have been documented so far in conjunction with frontotemporal lobar degeneration. Here we report the first case to our knowledge of a patient with frontotemporal lobar degeneration presenting a semantic variant subtype of primary progressive aphasia (svPPA) associated with repeatedly proven serum-GFAP autoantibodies. svPPA depicts one subtype of PPA along with the others, namely the non-fluent/agrammatic and logopenic PPA variant (5). These subtypes are differentiated clinically by their language and speech pattern and might be supported by neuroimaging evidence (5). These PPA subtypes reveal substantial clinical overlap that often makes them difficult to distinguish – a factor applying to PPA’s logopenic variant especially (6). A useful neuropsychological instrument [demonstrated recently (7) to distinguish svPPA from the non-fluent PPA variant] was to test patients’ naming capacity. Other novel diagnostic tools are having patients name animated objects: those with svPPA demonstrate a profound semantic deficit (8). The clinical spectrum of GFAP autoantibodies to date comprises patients with meningoencephalitis, headache, visual problems, often febrile temperature, abnormal movements (9), or hyponatremia (10). A recent study confirmed that the main clinical presentation associated with GFAP autoantibodies are subacute meningoencephalitis with dysfunction in cerebellar pathways (11). Myocloni and bulbar syndrome (12) as well as blindness (13) and vision loss (14) appear to be less frequent manifestations of GFAP-autoantibody disease. Thus, while GFAP autoantibody disease is heterogeneous, PPA has so far been reported to be associated with GFAP antibodies. Neuroinflammation’s potentially pathogenic role in PPA was recently highlighted in a study of 63 patients with sporadic PPA (15). There is thus evidence of inflammation in svPPA, but none yet of any association with GFAP antibodies.

## Case Report

We describe a 68-year-old woman who came to our memory outpatient clinic with word-finding difficulties, worsening memory, and problems with activities of daily life, over a year before presentation. Spontaneous speech presented with semantic paraphasia. Her psychopathological examination revealed a thought disorder comprising slowed, circuitous and discursive thinking, easily irritated and talking at cross purpose. Her drive was reduced, she was intermittently agitated and easily irritated. She had sometimes revealed impulsive behavior. Her neurological examination revealed ideatoric apraxia. Her internal examination was unremarkable. Her prior medical diagnoses encompassed ulcerous colitis, hypercholesterinemia, vitamin D deficiency, arterial hypertension and disturbed glucose tolerance. She had earlier been diagnosed with gastritis and amotio retinae on the right side. She is married and has two children. Her father was diagnosed with dementia in his old age. At first presentation the drugs she was taking were: gingko biloba 120 mg/d, mesalazine 1000 mg/d, metoprolol 23,75 mg/d, cholecalciferol 1000 IE orally, candesartan 16 mg/d, and atorvastatin 10 mg/d. Upon cognitive screening, her mini mental status examination (MMSE) score was 23/30 (**Table 1**).

**Table 1:** Laboratory assessment and cognitive data.

PARAMETER	FIRST PRESENTATION	FOLLOW UP
***CSF***		
Cells/μl(<5μg/L)	2	0
Albumin mg/L	319	325
IgG mg/L	43.9	39.8
IgA mg/ L	6.2	5.7
IgM mg/L	0.93	1.4
QAlb %	8	7.6
QIgG %	4	2 3.7
QIgA %	1.8	1.5
QIgM %	1.2	1.6
***Cell destruction marker CSF***		
Tau protein pg/ml (<450pg/ml)	473	495
P-Tau 181 pg/ml (<61pg/ml)	72	70
Aß42 pg/ml (>450pg/ml)	400	573
Aß40 pg/ml	8633	8706
Ratio Aß42/40 x10 (>0.5)	0.46	0.66
***Neural autoantibody***		
Autoantibody CSF	–	–
Autoantibody Serum	anti-GFAP (1:320+)	anti-GFAP (1:320+)
***Cognitive performance***		
MMSE (sum score)	23/30	–
CERAD Boston naming test	-5.8	–
CERAD semantic fluency	-3.9	–
CERAD phonemic fluency	-1.7	–
CERAD list learning (trials 1-3)	-3.6	–
CERAD list recall (savings)	-3.4	–
CERAD list recognition/discriminability	-1.8	–
CERAD figure recall (savings)	-2.7	–
CERAD figure copy	1.1	–
TMT part A	0.0	–
TMT division part B/A	0.9	–

Aß42, ß-amylod 42; Aß40, ß-amyloid 40; CERAD, The Consortium to Establish a Registry for Alzheimer's Disease; CSF, cerebrospinal fluid; GFAP, glial fibrillary acid protein; IgA, immunoglobulin A; IgG, immunoglobulin G; IgM, immunoglobulin M; P-Tau 181, phosphorylated tau protein 181; ratio Aß42/40, ratio ß-amyloid 42/40; QAlb, quotient albumin; QIgG, quotient immunoglobulin G; QIgA, quotient immunoglobulin A; QIgM, quotient immunoglobulin M; TMT, Trail Making Test. In the lab data, normal ranges are shown in brackets. In the neuropsychological data, z-values as normative data are depicted. Z-values <-1 mean performance below the normal range whereas z-values ≥-1 indicate performance within the normal range.

**Figure 1:**
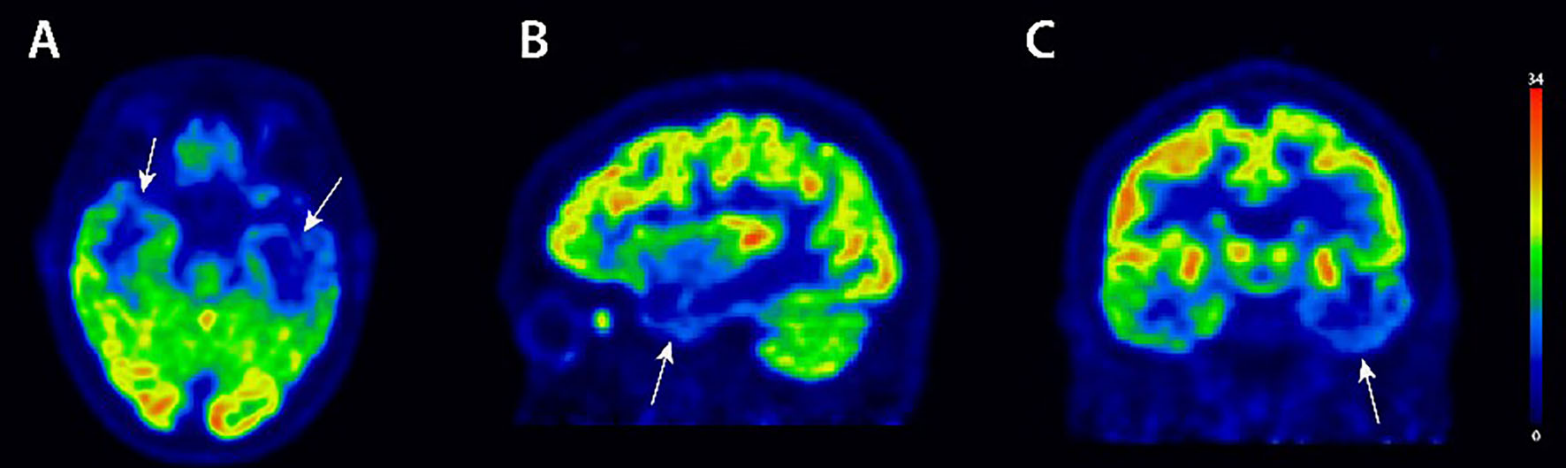
18F-FDG PET/CT imaging. 18F-FDG-PET images in axial **(A)**, sagittal **(B)** and coronal **(C)** view showing asymmetric hypometabolism in both temporal lobes (arrows), predominantly in the anterior left temporal lobe. 18F-FDG-PET/CT, 18F-fluorodeoxyglucose (18F-FDG) Positron Emission Tomography (PET)/computed tomography (CT) imaging.

Neuropsychological testing initially revealed impaired semantic and syntactic spontaneous speech. She also exhibited reduced semantic and phonemic word fluency, impaired confrontation naming and single-word comprehension. Additional anomalies were superficial dysgraphia, a pathological result in the clock drawing test and impaired figural and verbal memory (especially encoding/learning deficits). Speech repetition and speech motor function were normal. MRI revealed pronounced left medial temporal atrophy and hippocampal atrophy with signal enhancement in temporal T2/fluid attenuated inversion recovery (FLAIR) sequences. An 18F-fluorodeoxyglucose (18F-FDG) Positron Emission Tomography (PET)/computed tomography (CT) (18F-FDG-PET/CT) was performed later and revealed asymmetric hypometabolism involving both temporal lobes, predominantly the anterior left temporal lobe (**Figure 1**). Her electroencephalography (EEG) was unremarkable, displaying an alpha-beta ground rhythm without epileptic potentials. She underwent a lumbar puncture for differential diagnosis. Cerebrospinal fluid (CSF) analysis revealed repeatedly (at her baseline presentation and her follow-up two months later) elevated tau protein and phosphorylated tau protein (**Table 1**). However, the decreased ß-amyloid 42/40 (Aß42/40) ratio we had noted in her first CSF analysis was normal at follow-up 2 months later. We searched for these autoantibodies *via* immunoblots, immunofluorescence tests and cell-based assays in the Euroimmun laboratory: anti- α-amino-3-hydroxy-5-methyl-4-isoxazolepropionic acid receptor 1/2 (AMPAR1/2), amphiphysin, aquaporin 4, contactin associated protein 2 (CASPR2), CV2, dipeptidyl-peptidase–like protein-6 (DPPX), gamma aminobutyric acid B1/2 (GABAB1/2), glutamic acid decarboxylase (GAD65), HuD, leucin rich glioma inactivated protein 1 (LGI1), Ma1/2, NR1 subunit of the N-methyl-D-aspartate receptor (NMDAR), Ri, Ro, SOX1, TR, Yo and Zic4.

We detected anti-immunoglobulin G glial fibrillary acid protein (GFAP) antibodies in both serum analyses (done at the same time as her CSF analyses at baseline and follow-up) (1:320). Further blood investigations revealed slight hyperhomocysteinemia. We diagnosed a svPPA according the international consensus classification of Gorno-Tempini et al. (5), as these two essential features, namely impaired confrontation naming and single-word comprehension, were deficient. She fulfilled all four additional features such as spared speech production and repetition, impaired object knowledge, and surface dyslexia. In addition to these clinical features of the PPA’s semantic variant, she exhibited imaging characteristics such as anterior temporal hypometabolism in 18F-FDG-PET, supporting our diagnosis of an svPPA variant in an early stage. Nevertheless, an impaired figural memory is unusual in svPPA. However, an Alzheimer´s dementia (AD) is less likely as such severe speech problems are not typical of AD. A behavioral variant of frontotemporal dementia (bvFTD) is unlikely, as severe behavioral disturbances are missing apart from occasional impulsivity. Furthermore, her primary permanent speech disturbance argue against bvFTD. The other PPA subtypes are clinically unlikely and would have not been confirmed in the anterior hypometabolism in FDG-PET.

Having detected anti-GFAP antibodies could imply an autoimmune encephalitis. Accordingly, MRI suggested an encephalitis in the past with damage and atrophy in the hippocampi. However, her clinical phenotype and 18F-FDG-PET data are more likely to indicate a svPPA. Thus, it is likely that the GFAP antibodies are associated with svPPA. She has been given three cycles of steroids so far, but she has unfortunately failed to demonstrate any clinical improvement: her semantic paraphasia and word-finding difficulties persist. Long-term follow up is needed to monitor cognitive deficits that can be reversed or disease progression can be slowed at least.

## Discussion

Our case broadens on the one hand the clinical spectrum of GFAP astrocytopathy involving frontotemporal lobar degeneration in the form of svPPA as a phenotype. On the other hand, there is additional evidence that svPPA can be triggered or exacerbated by GFAP antibodies. The pathogenic role of GFAP antibodies remains incompletely understood although recent evidence indicates that CD8+T cells drive pathology in GFAP autoimmunity (16), moreover, the intracellular location of GFAP suggests that the role of GFAP antibodies is not pathogenic.

Our patient exhibited axonal neurodegeneration as well as elevated phosphorylated tau protein 181 and tau protein. However, it is not known whether GFAP antibodies lead to secondary neurodegeneration, or if they are an epiphenomenon unrelated to the neurodegeneration in our patient. Besides axonal degeneration, astrocytes might contribute to neurodegeneration. An animal study (17) demonstrated GFAP autoantibodies in cattle with bovine spongiform encephalopathy compared to healthy cattle, thus revealing a link between neurodegeneration and peripheral blood GFAP autoimmunity. A human study (18) suggested that GFAP autoantibodies of the IgG subtype are produced with one-week latency after the occurrence of a brain injury. Cell culture experiments (18) showed that GFAP autoantibodies can disrupt glial cell functioning by entering living astroglia cells, supporting the hypothesis of astroglial-derived neurodegeneration in our patient. In their study of 22 patients (19) with different clinical diagnosis ranging from meningoencephalomyelitis, encephalitis, ataxia to optic neuritis, GFAP autoantibodies were detected following a histopathological investigation of a leptomeningeal biopsy showing CD8+ T cells and macrophages. Their research group study reported that a combined T-cell and GFAP autoantibody dependent mechanism might induce neuroinflammation that could culminate in astroglial degeneration through still-unknown mechanisms. A recent study (20) reported the occurrence of black holes in the brain and spinal atrophy identified in patients with GFAP autoantibodies, underlying a neurodegenerative mechanism beyond autoimmunity. Thus, several mechanistic animal (17) and human studies (18–20) point towards astroglial damage in patients with GFAP autoantibodies.

An imaging marker was recently established to help diagnose svPPA, namely reduced metabolic activity in bilateral temporal lobes (21). Our patient partly fulfilled this type of metabolic pattern, as she revealed reduced bilateral metabolic activity in the temporal lobes. The same pattern of atrophy was also detected in structural neuroimaging, supporting the svPPA in our patient. MRI and 18F-FDG-PET data might also serve as valid markers of disease progression (21, 22). Recent findings suggest that the anterior temporal lobe degeneration in svPPA assessed *via* anterior temporal hypometabolism in FDG-PET, as in our patient, leads to a reorganized brain networks with dysfunctional semantic representations compensated by augmented perception (23). The cerebral metabolism is specific for each PPA variant, and correlates strongly with the PPA subtype (24), supporting the usefulness of FDG-PET for the differential diagnosis of PPA subtypes, as was the case for our patient. FDG-PET is known to be valuable for the early diagnosis of PPA subtypes (25) detecting synaptic dysfunction that appears earlier than atrophy (26). However, the MRI pattern did not clearly support our clinical diagnosis of svPPA, although the MRI pattern of anterior temporal lobe atrophy is known to be highly specific and sensitive among the other PPA subtypes (27).

In the absence of therapeutic options for frontotemporal dementia, our case reveals the need for neural autoantibody testing in such patients. We wish to draw attention to the recently reported case of patient with GFAP antibody-associated progressive dementia (28) who responded rapidly (and so well) to immunotherapy that led to a stabilization without further deterioration of her dementia.

There is no data that we are aware of on the efficacy of immunotherapy in such cases. We suspected an autoimmune etiology because of her repeatedly detected serum GFAP antibodies, and applied high-dose intravenous steroids. Previous trial data have suggested high responsiveness to steroids from patients with autoimmune dementia and autoantibody-associated cognitive impairment (29–32), which is why we presumed a benefit by applying steroids. She has not shown any neuropsychological improvement so far, but has only been given one cycle until now. Before her responsiveness can be assessed, we believe she must complete three more cycles of steroids. Large-scale and longitudinal studies are required to evaluate the benefit of immunotherapy in patients with neural autoantibody-associated dementia and subtypes like frontotemporal dementia. Our patient’s immunopathology is still unclear, thus at present, we can only speculate that her dementia has an immunopathological basis. However, we do think that frontotemporal dementia and autoimmune diseases are interlinked. There is recent evidence that svPPA and frontotemporal dementia with progranulin mutation carriers are associated with non-thyroid autoimmune disease more often than AD patients or normal subjects are (33). This link is further supported by recent research that revealed some FTD related genes such as C9orf72 are also involved in neuroinflammatory processes [(34); for review see (35)]. It would therefore be worthwhile to investigate whether certain types of autoantibodies such as GFAP in svPPA reflect an underlying neuroinflammation responsible for initiating or maintaining neurodegeneration. The other conceivable possibility is that GFAP-associated dementia is an individual disease type that differs from classical svPPA. However, as its phenotype is so similar to svPPA that svPPA associated with GFAP antibody is the patient’s most likely diagnosis, while the pathogenic relevance of the GFAP antibodies in this type of dementia remains thus far unclear.

Limitations of our report concern the serum (and not CSF-proof) of GFAP autoantibodies, as they are less accurate indicators of an autoimmune origin of svPPA. However, the repeated proofs of serum GFAP antibodies argue against a casual association. Furthermore, the diagnostic criteria of svPPA are clearly supported by the clinical and neuroimaging evidence we collected, so that there can be no doubt about the diagnosis. The strength of our study is the extensive neuropsychological investigation combined with neuroimaging techniques and our assessment of anti-neural autoantibodies.

Taken together, our report expands our knowledge of subtyping svPPA, and elaborates upon the disease entity of a frontotemporal dementia potentially caused by neuroinflammation.

## Data Availability Statement

The original contributions presented in the study are included in the article. Further inquiries can be directed to the corresponding author.

## Ethics Statement

Ethical review and approval was not required for the study on human participants in accordance with the local legislation and institutional requirements. The patients provided her written informed consent to participate in this study. Written informed consent was obtained from the individual for the publication of any potentially identifiable images or data included in this article.

## Author Contributions

NH, CBo and CBa wrote the manuscript. JW, KR, and WS revised the manuscript for important intellectual content. All authors contributed to the article and approved the submitted version.

## Funding

Funding is derived from the Open access fund of the University of Göttingen.

## Conflict of Interest

The authors declare that the research was conducted in the absence of any commercial or financial relationships that could be construed as a potential conflict of interest.

## Publisher’s Note

All claims expressed in this article are solely those of the authors and do not necessarily represent those of their affiliated organizations, or those of the publisher, the editors and the reviewers. Any product that may be evaluated in this article, or claim that may be made by its manufacturer, is not guaranteed or endorsed by the publisher.
